# Total hip arthroplasty compared to bipolar and unipolar hemiarthroplasty for displaced hip fractures in the elderly: a Bayesian network meta-analysis

**DOI:** 10.1007/s00068-022-01905-2

**Published:** 2022-02-19

**Authors:** Filippo Migliorini, Nicola Maffulli, Mira Trivellas, Jörg Eschweiler, Frank Hildebrand, Marcel Betsch

**Affiliations:** 1 Department of Orthopaedic, Trauma, and Reconstructive Surgery, RWTH University Hospital, Pauwelsstraße 30, 52074 Aachen, Germany; 2 Department of Medicine, Surgery and Dentistry, University of Salerno, Via S. Allende, 84081 Baronissi, SA Italy; 3 Queen Mary University of London, Barts and the London School of Medicine and Dentistry, Centre for Sports and Exercise Medicine, Mile End Hospital, 275 Bancroft Road, London, E1 4DG England; 4 School of Pharmacy and Bioengineering, Keele University Faculty of Medicine, Thornburrow Drive, Stoke on Trent, England; 5 Department of Orthopedics and Trauma Surgery, David Geffen School of Medicine at UCLA, Los Angeles, CA USA; 6 Department of Orthopedics and Trauma Surgery, University Clinic Mannheim, 68167 Mannheim, Germany

**Keywords:** Femoral fractures, Elderly, Hip arthroplasty, Hemiarthroplasty

## Abstract

**Purpose:**

Displaced femoral neck fractures (FNF) usually require surgical treatment with either a total hip arthroplasty (THA), unipolar hemiarthroplasty (U-HHA), or bipolar hemiarthroplasty (B-HHA). However, there is still controversy regarding the optimal implant. This network meta-analysis compared the outcomes and complication rates of THA versus B-HHA and versus U-HHA in elderly patients with FNF.

**Material and methods:**

This study was conducted according to the PRISMA extension statement for reporting of systematic reviews, and incorporated network meta-analyses of health care interventions. The literature search was performed in September 2020. All randomized clinical trials comparing two or more of the index surgical interventions for displaced FNF in the elderly were eligible for inclusion. For the Bayesian network meta-analysis, the standardized mean difference (SMD) and Log Odd Ratio (LOR) were used.

**Results:**

Data from 24 RCTs (2808 procedures) were analysed. The mean follow-up was 33.8 months. The THA group had the longest surgical time (SMD 85.74) and the greatest Harris Hip Score (SMD − 17.31). THA scored similarly in terms of mortality (LOR 3.89), but had lower rates of revision surgeries (LOR 2.24), higher rates of dislocations (LOR 2.60), and lower rates of acetabular erosion (LOR − 0.02). Cementless implants required a shorter surgical duration (− 18.05 min; *P* = 0.03). Mortality was positively associated with acetabular erosion (*P* = 0.006), female gender (*P* = 0.007), revision (*P* < 0.0001).

**Conclusion:**

THA led to the highest Harris Hip scores and lowest rate of revision surgery compared to B-HHA and U-HHA. However, B-HHA had the lowest dislocation rate when compared with U-HHA and THA. No significant differences in functional outcomes and complication rates were found between cemented and uncemented implants; however, a tendency for lower mortality, revision and dislocation rates in cemented implants was evidenced.

**Level of evidence:**

I, Bayesian network meta-analysis of RCTs.

## Introduction

Hip fractures are common in the elderly [1, 2]. Most of these fractures are a consequence of trauma and osteoporosis [3, 4]*.* Annually, around 1.5 million hip fractures occur worldwide. These fractures are expected to increase to more than 6 million by 2050, given demographic changes and the increasing incidence in developing countries [5–7]. In the elderly, hip fractures may lead to significant mortality and morbidity, with impaired mobility and inherent loss of independence [8–11]. Given their high incidence and associated detrimental effects on patient lives, hip fractures are considered a global health and economic burden, with a cost of 13 billion US dollars per year [3].

Displaced and unstable femoral neck fractures (FNF) are most common, and require early surgical intervention with either a total hip arthroplasty (THA), unipolar hemiarthroplasty (U-HHA), or bipolar hemiarthroplasty (B-HHA) [12–17]. Surgery in these patients is undertaken to facilitate nursing, and provide timely pain relief, rapid mobilization, and accelerated rehabilitation [18, 19].

HHA is the preferred treatment option for displaced FNF, as it is faster and leads to satisfactory function and performance in the elderly [20–23]. In HHA, surgeons can choose to use a unipolar or bipolar femoral head, using the latter to maximize the longevity of the implant. B-HHA uses an additional inner bearing between the stem and the femoral head to decrease the rate of acetabular erosion and protrusion by maintaining joint stability and improving joint function [24, 25]. Additionally, the surgeon can choose to use a press-fit or cemented femoral stem. Potential benefits of cemented stems are a reduced risk of periprosthetic fractures and improved bony fixation in elderly patients with osteoporosis and poor potential for bony ingrowth required in press-fit techniques [26, 27]. Possible disadvantages of cemented stems are the risks associated with increased surgical time and increased perioperative mortality from cardio-vascular complications [28, 29]. Furthermore, the cementing technique requires consistency and diligence to allow the cement mantle to cure appropriately and have the best chances for suitable longevity. Cement is at its strongest on the day of the operation, and the strength will only decrease with time and forces applied to the implant [30]. Implant-specific advantages and disadvantages add to the variability among orthopaedic surgeons’ choice of the implant to treat a displaced FNF. The use of bone cement has been associated with greater intraoperative morbidity; however, this can be reduced by intramedullary lavage and modern cementing techniques [31, 32]. Uncemented stems produce greater thigh pain and poorer overall function [33–35], contrary to cemented stems [36–38]. A Bayesian network meta-analysis was performed to compare the outcomes and complication rates of total hip arthroplasty versus bipolar hemiarthroplasty and versus unipolar hemiarthroplasty in the management of displaced femoral neck fractures in the elderly.

## Material and methods

### Search strategy

This Bayesian network meta-analysis was conducted according to the PRISMA extension statement for reporting of systematic reviews incorporating network meta-analyses of health care interventions [39]. The PICO algorithm guided the preliminary search:

- P (population): displaced femoral neck fractures;
- I (intervention): hip arthroplasty;
- C (comparison): unipolar hemiarthroplasty, bipolar hemiarthroplasty, total arthroplasty;
- O (outcomes): hospitalization, surgical duration, Harris hip score, complications, mortality.

### Data source and extraction

Two authors independently (**;**) accessed the main online databases in September 2020: PubMed, Google scholar, EMBASE, and Scopus. The following keywords were used in combination: *hip, femoral, fractures, displaced, elderly, total, therapy, treatment, surgery, surgical, arthroplasty, replacement, prosthesis, hemiarthroplasty, unipolar, bipolar, complications, mortality, rate, death, survivorship, womac, harris hip, index, scale, score, revision, dislocations*. The same authors independently performed the initial screening. If the title and abstract matched the topic, the full text of the article was analysed. A cross-reference of the bibliographies was also conducted. Disagreement was debated and resolved by a third author (MB).

### Eligibility criteria

All randomized clinical trials (RCTs) comparing two or more surgical treatments between total arthroplasty, bipolar, or unipolar hemiarthroplasty for displaced femoral fractures were considered for inclusion. According to the authors’ language capabilities, articles in English, French, German, Italian, Portuguese and Spanish were eligible. Only studies with level I or II evidence according to the Oxford Centre of Evidence-Based Medicine [40] were eligible for analysis. Only articles with patients older than 60 years were considered for inclusion. Every type of implant, surgical approach, and incision length were considered for inclusion. Studies evaluating navigation systems were included as well. Both cemented and uncemented implants were included. Studies concerning revision settings were excluded. Studies evaluating the addition of adjuvants, such as stem cells, PRP, or any other substances, in these procedures were excluded. Reports, reviews, letters, comments, registry studies, and editorials were excluded. Animal, biomechanics, and cadaveric studies were excluded. Only studies which clearly stated the type of implant and reported the results in a separate fashion were included. Only articles reporting quantitative data on the outcomes of interest were considered for inclusion. Incomplete data of the outcomes of interest warranted exclusion from this study. Disagreement between the authors were mutually debated and resolved by a third, senior author (**).

### Outcomes of interest

Two authors independently (**;**) performed data extraction. Study specifics (author, year, type of study, follow-up term) and patients baseline demographic information were collected (number of procedures, mean age, gender). The outcomes of interest were: hospitalization length, surgical duration, Harris hip score, complications (acetabular erosion, dislocations, revisions), and mortality.

### Methodology quality assessment

The methodology quality assessment was performed by two authors (**;**) using the risk of bias summary from the Review Manager software (The Nordic Cochrane Collaboration, Copenhagen). The biases evaluated in the analysis were: selection, detection, attrition, reporting, and other sources.

### Statistical analysis

The statistical analyses were performed by one author (**). Baseline comparability was assessed through the IBM SPSS software. The analysis of variance (ANOVA) was used for analysis, with *P* values $\ge$ 0.5 considered satisfactory. The STATA Software/MP, Version 14.1 (StataCorporation, College Station, Texas, USA) was used for the Bayesian network analyses, as per routine for Bayesian hierarchical random-effects model analysis. The inverse variance method was used for the analysis of continuous and binary variables, with a standardized mean difference (STD) and Log Odd Ratio (LOR) effect measures. Confidence (CI) and percentile (PrI) intervals were set at 95%. The overall inconsistency was evaluated through the equation for global linearity via the Wald test. If the *P* value was > 0.5, the null hypothesis could not be rejected, and the consistency assumption could be accepted at the overall level of each treatment. Edge plot, interval plots, and funnel plots were used to evaluate the data. A multivariate analysis was performed to correlate baseline data and surgical outcomes. For analyses, multiple pairwise correlations with the Pearson Product-Moment Correlation Coefficient (*r*) were performed. According to the Cauchy–Schwarz inequality, the final effect ranked between + 1 (positive linear correlation) and − 1 (negative linear correlation). Values of 0.1 <| *r* |< 0.3, 0.3 <| *r* |< 0.5, and | *r* |> 0.5 were considered to have small, medium, and moderate correlation, respectively. The test for overall significance was performed through the χ^2^ test, with values of *P* > 0.05 considered statistically significant. For the statistically significant correlations, a linear regression analysis was performed, and Added-Variable plots were generated.

## Results

### Search result

**Fig. 1:**
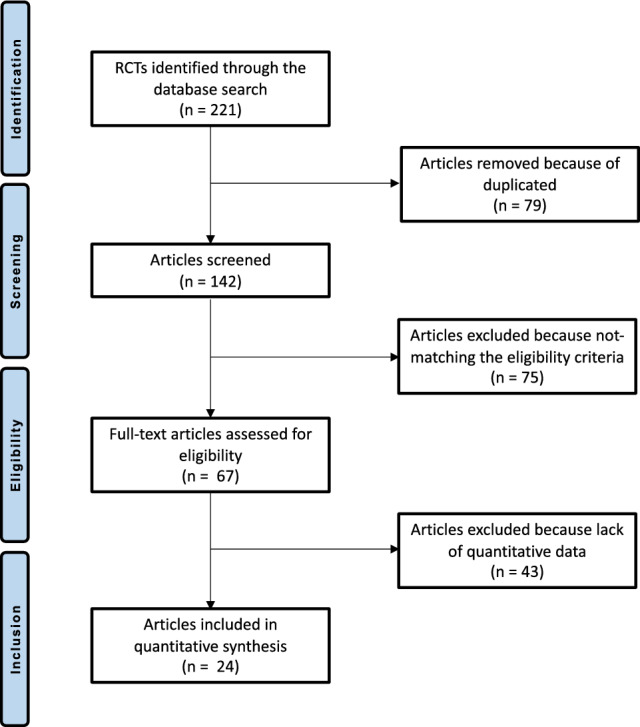
Flow chart of the literature search

The literature search resulted in 1511 articles, of which 221 were RCTs. 79 articles were excluded because of duplication. Eleven articles were excluded because of language limitations, and 51 did not match the type of study. Another 13 studies did not match the eligibility criteria. A total of 43 articles were excluded because they did not report quantitative data for the outcomes of interest. This left 24 RCTs to analyse for the present study. The literature search results are shown in Fig. 1.

### Methodological quality assessment

**Fig. 2:**
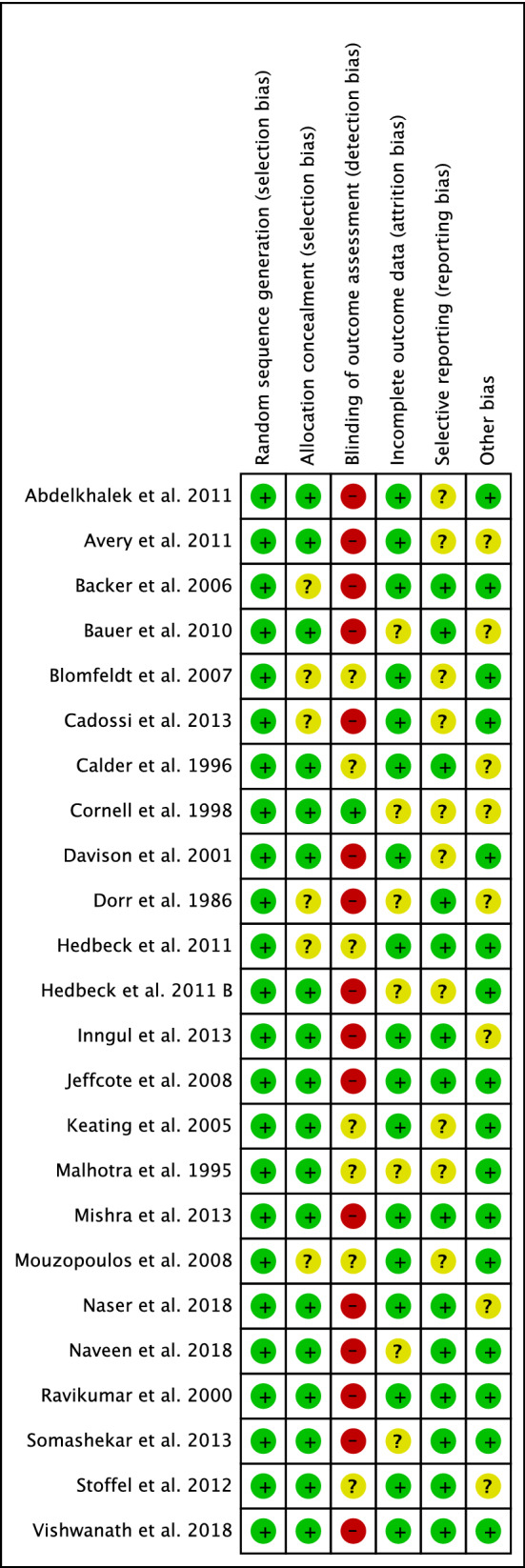
Methodological quality assessment

The risk of bias summary was a limitation of the present study given the inherent inclusion and exclusion criteria of each RCT. The risk of selection bias was low. The risk of detection bias was moderate, as many articles did not use any blinding. The risk of attrition, reporting, and other bias was low to moderate. Overall, the quality of the methodological assessment was good. The Cochrane risk of bias summary is shown in Fig. 2.

### Patient demographics

Data from 2808 procedures were retrieved. The mean follow-up was 33.8 ± 34.3 months. The mean age of the patients at baseline was 77.2 ± 6.7 years, and 71% (1994 of 2808 patients) were women. Between THA, U-HHA, and B-HHA cohorts, the ANOVA test found good baseline comparability in age (*P* = 0.8) and gender (*P* = 0.7). Patient demographic is shown in Table 1.

**Table 1:** Generalities and patients baseline of the included studies

Author	Follow up (*months*)	Treatment	Cementation	Procedures (*n*)	Female Gender	Mean Age (*years*)
Abdelkhalek et al. [55]	52.80	U-HHA		25	68.0	63.5
		B-HHA		25	68.0	63.5
Avery et al. [56]	106	THA	Cemented	21		80.0
		B-HHA	Cemented	13		84.0
Backer et al. [57]	36	THA	Cemented	40	80.0	74.2
		B-HHA	Cemented	41	78.1	75.8
Bauer et al. [58]	6.00	U-HHA	Cemented	206	80.0	85.0
		B-HHA	Cemented	97	79.0	78.0
Blomfeldt et al. [59]	12	THA	Cemented	60	78.0	80.1
		B-HHA	Cemented	60	90.0	80.7
Cadossi et al. [60]	29	THA	Cemented	47	32.0	84.2
		B-HHA	Mixed	49	19.0	82.3
Calder et al. [61]	21.50	U-HHA	Cemented	132	86.4	85.0
		B-HHA	Cemented	118	85.6	85.0
Cornell et al [62]	6.00	U-HHA	Cemented	15	73.3	77.6
		B-HHA	Cemented	33	75.8	78.0
Davison et al. [63]	36.00	U-HHA	Cemented	97	74.2	75.0
		B-HHA	Cemented	90	78.9	76.0
Dorr et al. [35]	48.00	THA	Cemented	39	59.0	69.0
		B-HHA	Mixed	50	70.0	
Hedbeck et al. [64]	48	THA	Cemented	60	78.0	80.5
		B-HHA	Cemented	60	90.0	80.7
Hedbeck et al. [65]	12.00	U-HHA	Cemented	60	82.0	87.4
		B-HHA	Cemented	60	70.0	85.5
Inngul et al. [43]	48.00	U-HHA	Cemented	60	82.0	87.4
		B-HHA	Cemented	60	70.0	85.5
Jeffcote et al. [66]	24.00	U-HHA	Cemented	27	77.7	81.4
		B-HHA	Cemented	24	75.0	80.1
Keating et al. [67]	24	THA	Cemented	69	75.0	75.2
		B-HHA	Cemented	69	78.0	75.0
Malhotra et al. [68]	24.50	U-HHA	Uncemented	36	44.5	68.0
		B-HHA	Uncemented	32	43.7	65.0
Mishra et al. [69]	12.00	U-HHA		20	68.0	67.0
		B-HHA		20	68.0	67.0
Mouzopoulos et al. [70]	48	THA	Cemented	37	75.7	73.1
		B-HHA		34	70.6	74.2
Naser et al. [71]	12.00	U-HHA		70	68.0	
		B-HHA		70	68.0	
Naveen et al. [72]	12.00	U-HHA		50	56.0	76.8
		B-HHA		50	56.0	76.8
Ravikumar et al. [73]	156	THA	Cemented	89	90.0	81.0
		U-HHA	Uncemented	91	90.0	82.1
Somashekar et al. [74]	12.00	U-HHA	Uncemented	20	47.6	75.6
		B-HHA	Uncemented	21	85.0	67.3
Stoffel et al. [75]	12.00	U-HHA	Cemented	126	72.0	81.9
		B-HHA	Cemented	133	72.0	82.9
Vishwanath et al. [76]	12.00	U-HHA		50	62.0	70.4
		B-HHA		52	62.0	69.1

### Network comparisons

**Fig. 3:**
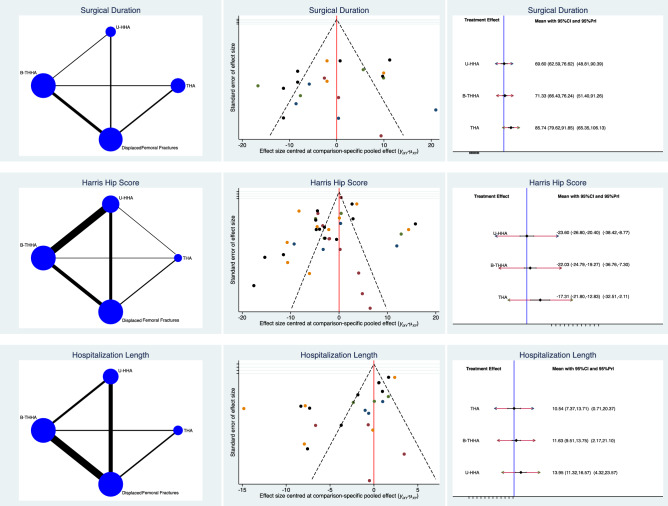
Results of the network comparison: surgical duration, HHS, hospitalization

The THA group had the longest surgical time (SMD 85.74; 95% CI 79.62–91.85), while the U-HHA (SMD 69.60; 95% CI 62.59–76.62) and B-HHA were similar in surgical duration (SMD 71.33; 95% CI 66.43–76.24). The THA group had the highest HHS (SMD − 17.31; 95% CI − 21.80 to − 12.83), while the U-HHA (SMD − 23.60; 95% CI − 26.80 to − 20.40) and B-HHA had similar scores (SMD − 22.03; 95% CI − 24.79 to − 19.27). Edge, funnel, and interval plots of the comparisons concerning the surgical duration, HHS, hospitalization are shown in Fig. 3.

**Fig. 4:**
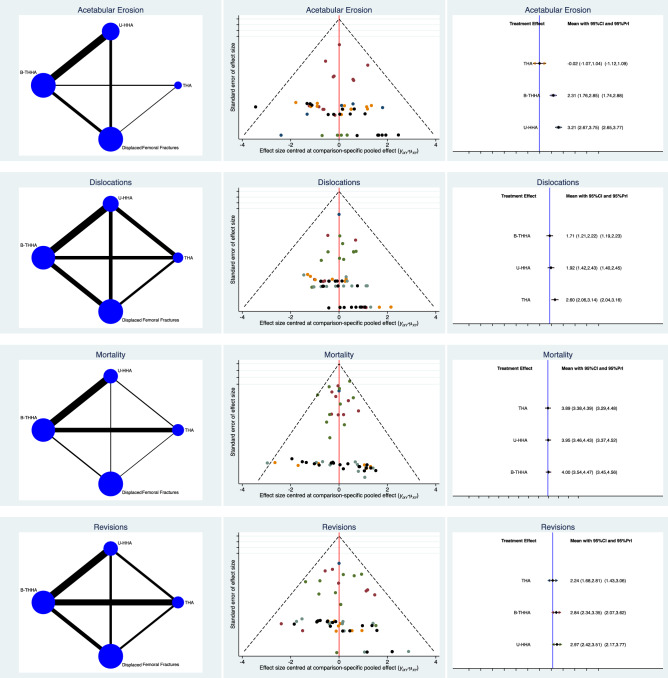
Results of the network comparison: complications

THA scored similarly in terms of mortality (LOR 3.89; 95% CI 4.43) and B-HHA (LOR 4.00; 95% CI 3.54–4.47). Patients who underwent a THA had a lower rate of revision surgeries (LOR 2.24; 95% CI 1.68–2.81), compared to those who had a B-HHA (LOR 2.84; 95% CI 2.34–3.35) or a U-HHA (LOR 2.97; 95% CI 2.42–3.51). THA was associated with a higher rate of dislocation (LOR 2.60; 95% CI 2.06–3.14), followed by U-HHA (LOR 1.92; 95% CI 1.42–2.43), and B-HHA had the fewest (LOR 1.71; 95% CI 1.21–2.22). The THA group had the lowest rate of acetabular erosion (LOR − 0.02; 95% CI − 1.07 to 1.04), followed by B-HHA, (LOR 2.31; 95% CI 1.76–2.85), whereas the U-HHA group had the highest (LOR 3.21; 95% CI 2.67–3.75). Using the equation for global linearity for the endpoints analysed, hospitalization length was considerably inconsistent (*P* = 0.001) and, therefore, not reliable. Edge, funnel, and interval plots of the comparisons concerning the complication rates are shown in Fig. 4.

### Cemented versus uncemented implants

Implanting uncemented components required a shorter surgical duration (− 18.05 min; *P* = 0.03). Otherwise, no statistically significant differences were found between implants or fixation technique in regards to HHS, hospitalization length, acetabular erosion, mortality rate, revision surgeries, and rate of dislocations (Table 2).

**Table 2:** Comparisons of cemented versus uncemented implants

Endpoint	Uncemented (*n* = 200)		Cemented (*n* = 2043)		*P*
	Mean	SD	Mean	SD	
Harris Hip Score	82.99	4.5	75.04	8.3	0.1
Surgical duration (minutes)	60.00	7.5	78.05	11.6	0.03
Hospital length of stay (days)	17.67	0.6	13.29	5.2	0.1
Acetabular erosion (5)	4.60	8.1	3.89	5.2	0.4
Mortality (%)	27.67	43.6	16.86	17.7	0.2
Revisions (%)	5.20	9.5	2.96	2.3	0.1
Dislocations (%)	3.00	5.0	2.21	3.5	0.3

### Multivariate analysis

**Fig. 5:**
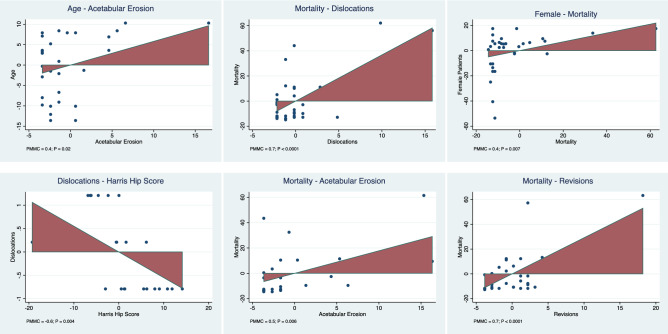
Added-variable plots of the statistically significant regressions

There was evidence of a statistically significant positive association between age and acetabular erosion (*r* = 0.4; *P* = 0.02). There was a statistically significant negative association between HHS and dislocations (*r* = -0.6; *P* = 0.004). Mortality was positively associated with acetabular erosion (*r* = 0.5; *P* = 0.006), female gender (*r* = 0.4; *P* = 0.007), and revision surgery (*r* = 0.7; *P* < 0.0001). No other statistically significant associations were found. The added-variable plot of each linear meta-regression is shown in Fig. 5.

## Discussion

There is still controversy on the optimal implants for the management of patients with a displaced FNF. The present Bayesian network meta-analysis compared treatment options for displaced FNF in elderly patients based on outcomes and complications. A total of 24 RCTs were included in this study, with a mean follow-up of 33.8 months. Overall, THA was associated with higher HHS, lower rates of revision surgery, and lower rates of acetabular erosion. However, patients with a displaced FNF who undergo a THA are at risk of considerably higher rates of dislocation. No differences were found in terms of mortality rates between the different implants, and no differences in mortality rates were found between cemented or uncemented stems; however, we found a tendency for lower mortality, revision and dislocation rates in cemented implants. Based on a multivariate analysis, older patients are more prone to acetabular erosion, while female gender, advanced age and revision surgeries were positively associated with a higher mortality rate. As expected, THA and cementing lead to a significant increase in surgical duration, since both procedures include additional surgical steps.

The HHS, which ranges from 0 to 100 points, is frequently used to evaluate clinical outcomes after THA and HHA measuring function, pain, deformity, and range of motion. To compare postoperative hip function in the present network meta-analysis, we compared the HHS for the three surgical techniques studied. A recent meta-analysis found no significant differences in HHS between THA and B-HHA; however, there was a trend towards higher HHS in the THA group [41]. This was different compared to our results, and the findings of Burgers et al. who found significantly higher HHS in patients treated with THA [42].

THA leads to a significant decrease in acetabular erosion and, therefore, a lower rate of revision surgery for this ailment when compared to B-HHA and U-HHA. We found a higher rate of acetabular erosion in U-HHA than in B-HHA; however, no differences in revision surgery between the two techniques were found. This was previously reported: B-HHA could lead to increased later onset of acetabular erosion compared to U-HHA, and, consequently, the re-operation rate in B-HHA was expected to be at least equivalent to that of U-HHA [43, 44].

In our analysis, the length of hospital stay between the three techniques was remarkably inconsistent, and was, therefore, considered not reliable. This was similar to the analysis by Wang et al. in [41], comparing B-HHA with THA in 1014 patients. Woon et al., who analysed data from the US National Hospital Discharge Survey, evidenced high heterogeneity between different regions and hospital sizes. Conversely, they showed a decrease in hospitalization length in patients treated with HHA [45].

Our analysis showed that the mortality rate after THA, B-HHA, and U-HHA was similar regardless of the technique used: the type of surgical treatment does not significantly affect mortality. A recent meta-analysis, which included seven studies which assessed the one-year mortality rate, showed no significant differences between the THA and B-HHA groups [41]. Zhang et al., in 2017, also showed no significant differences in mortality rates among different treatment options for displaced FNF [46]. Finally, a meta-analysis by Burgers et al. included 816 patients, and the one-year mortality rate was 13% in the THA group versus 15% in the HHA group, with no statistically significant differences [42].

Another major complication following THA or HHA for the treatment of displaced FNF is post-operative dislocation. The use of B-HHA leads to the lowest dislocation rate followed by U-HHA, while THA had the highest rate of dislocation. This was consistent with Zhang et al.’s findings: THA had the highest dislocation rate and B-HHA had the lowest dislocation rate [46]. The soft tissue releases and acetabular bone stock removal that are required for implantation of the acetabular cup potentially destabilizes the hip joint [46] Furthermore, without the need to fit the prosthetic head into an acetabular component, surgeons can place a larger femoral head, which decreases the risk of dislocation and impingement [47, 48]. Burgers et al. also reported lower dislocation rates using HHA in over 800 patients treated with THA or HHA for displaced FNF [42]. Similar results were reported by Yu et al. and Wang et al., who found significantly lower dislocation rates in patients undergoing HHA [41, 49].

Both cemented and uncemented fixation of the femoral stem are currently used in THA and HHA, and controversy still exists regarding the ideal method. As expected, our results showed a shorter operation time with uncemented implants; however, there were no statistically significant differences in functional outcomes or complication rates between the techniques with a tendency for lower mortality, revision and dislocation rates in cemented implants. This was consistent with the work by Ahn et al., who showed that post-operative mortality rates, overall complications, and pain were similar between the two cohorts [50]. In 2020, Li et al. performed a meta-analysis comparing the outcomes of patients treated with cemented HHA versus uncemented HHA for displaced FNF. They identified no differences in HHS scores, mortality rates at 12 months, hospital stay, or blood loss between the two fixation techniques [51]. They highlighted a higher rate of pulmonary embolism following cemented HHA. However, a Cochrane analysis from 2010 comparing femoral fixation techniques, independent of the prosthetic design, reported reduced post-operative pain and better mobility using a cemented femoral stem [52]. Kumar et al. [53], in a recent systematic review on 2819 procedures, found that cemented implants were associated with a lower risk of intra- and postoperative rate of fractures. We believe that additional prospective randomized trials with larger patient populations are necessary to further evaluate differences in fixation techniques.

This study does have several limitations. Only 24 of the 142 original RCTs identified in our literature search met our inclusion criteria. Several RCTs compared the outcomes of THA versus HHA without clarifying whether a bipolar or monopolar implant was used, or did not report data separately. Additionally, only studies with a level of evidence of I or II were included, which decreased the number of available studies; however, this improved the quality of data. Another limitation is represented by the heterogeneous type of implants used in each group, which increases the selection bias. Furthermore, outcome parameters can vary significantly between different studies, which made it difficult to include additional parameters to evaluate hip function. We acknowledge that, even if no significant inconsistency has been detected, the endpoint length of the hospitalization stay may be strongly influenced by the health system and the different health insurances. A major complication following surgical treatment of femoral neck fractures is a periprosthetic fracture. However, in the current analysis, because of the lack of currently available data, we were not able to analyse the risk of periprosthetic fractures: this is a limitation of this current work. Konow et al., in 2021, found a higher periprosthetic fracture risk in uncemented and collarless femoral components compared to cemented and collared prothesis [54]. Given these limitations, the results from the present study must be interpreted with caution.

## Conclusion

In conclusion, we performed a comprehensive network meta-analysis comparing current treatment options for displaced FNF in elderly patients. THA leads to the highest HHS scores with the lowest rate of revision surgery. However, B-HHA has the lowest dislocation rate when compared with U-HHA and THA. All three techniques showed similar mortality rates. No significant functional differences and no differences in complication rates were detected between cemented and uncemented implants; however, there was a tendency for lower mortality, revision and dislocation rates in cemented implants. Our results must be interpreted within the limitations of the present study.

## Data Availability

The data underlying this article are available in the article and in its online supplementary material.
